# Trends in the use of Japanese herbal Kampo medicine in inpatients with cancer: a 14-year nationwide analysis

**DOI:** 10.1007/s10147-025-02866-3

**Published:** 2025-09-22

**Authors:** Chikako Iwai, Takaaki Konishi, Shotaro Aso, Hiroki Matsui, Kiyohide Fushimi, Hideo Yasunaga

**Affiliations:** 1 Department of Clinical Epidemiology and Health Economics, School of Public Health, The University of Tokyo, 7-3-1 Hongo, Bunkyo-Ku, Tokyo, 113-0033 Japan; 2 Department of Breast and Endocrine Surgery, Graduate School of Medicine, The University of Tokyo, Tokyo, Japan; 3 Department of Health Services Research, Graduate School of Medicine, The University of Tokyo, Tokyo, Japan; 4 Department of Health Policy and Informatics, Institute of Science Tokyo Graduate School of Medical and Dental Sciences, Tokyo, Japan

**Keywords:** Breast cancer, Colorectal cancer, Gastric cancer, Japanese herbal medicine, Lung cancer, Prostate cancer

## Abstract

**Background:**

Kampo medicine (Japanese herbal medicine) is often used to manage the side effects of cancer treatment and improve quality of life. However, nationwide trends in Kampo use among inpatients with cancer in Japan remain poorly understood. This study aimed to examine the temporal trends and clinical practice patterns of Kampo prescriptions among inpatients with cancer between 2010 and 2023, with a focus on their use in acute or severe conditions.

**Methods:**

This retrospective cohort study used the Japanese Diagnosis Procedure Combination database, including over 6.8 million hospitalizations for five major cancer types (breast, colorectal, gastric, lung, and prostate)between July 2010 and March 2023. Kampo use was identified from in-hospital prescription data. Prescription trends and patient characteristics were analyzed.

**Results:**

Kampo medicines were prescribed in 13.6% of hospitalizations, more commonly among older adults and patients with colorectal cancer. The overall prescription proportion increased from 2010 to 2017 before plateauing. The top five prescribed Kampo medicines were Dai-ken-chu-to, Gosha-jinki-gan, Rikkunshi-to, Shakuyaku-kanzo-to, and Hange-shashin-to. Prescription patterns varied by age group, cancer type, cancer stage, disease status, and cause of hospitalization. Dai-ken-chu-to use shifted from postoperative to chronic care, while Gosha-jinki-gan prescriptions gradually declined.

**Conclusions:**

Kampo prescription patterns among inpatients with cancer have changed over the past fourteen years, reflecting changes in patient demographics and treatment strategies. Kampo medicines appear to be selectively used as supportive care tailored to specific clinical situations. These findings highlight the evolving role of traditional medicine in modern cancer care in Japan.

## Introduction

Cancer is a serious global health issue, with approximately 20 million new cases and 10.8 million deaths reported annually worldwide [1]. In Japan, approximately 390,000 people die from cancer annually, accounting for 27.4% of all deaths [2]. Cancer also poses a heavy socioeconomic burden, accounting for approximately 11% of total medical expenses [3]. Improving quality of life and minimizing treatment-related side effects are key priorities in the clinical management of patients with cancer [4–6].

Kampo medicine, Japanese herbal medicine, is expected to reduce the side effects of chemotherapy and radiotherapy and improve the quality of life in patients with cancer [7–11]. Although its efficacy and empirical applicability have been recognized [9], detailed information on recent treatment patterns involving Kampo medicine in cancer care remains insufficient. Kampo medicines are often prescribed to outpatients and can be used continuously across outpatient and inpatient settings. Kampo medicines are also used in various contexts, including home-based palliative care for patients who are terminally ill. To our knowledge, there has been no study investigating the use of Kampo medicines in patients with cancer requiring inpatient care. Therefore, this focused on the use of Kampo medicines in acute or severe conditions in patients with cancer. Understanding nationwide trends and clinical practices related to Kampo medicine is crucial for optimizing cancer treatment strategies**.** Furthermore, such insights could contribute to the establishment of evidence-based medical practice**.**

This study aimed to evaluate recent trends in the use of Kampo medicine among hospitalized patients with cancer in Japan, focusing on the most common cancer types (breast, colorectal, gastric, lung, and prostate) between 2010 and 2023 using a nationwide database.

## Patients and methods

### Data source

This nationwide retrospective cohort study used the Japanese Diagnosis Procedure Combination (DPC) database. Data use was approved based on research purposes, with access granted as a member of the corresponding research group. This database includes discharge summaries and administrative claims data, covering approximately 8 million hospital admissions from over 1,200 hospitals across Japan. These admissions represent nearly half of all admissions to acute care hospitals in the country [12]. While participation is mandatory for all 82 academic hospitals in Japan, community hospitals participate on a voluntary basis.

The database contains patient-related information [13], such as unique hospital identifiers, demographic data (age and sex), smoking history (including current and former smokers), body mass index at admission, and activities of daily living at admission. It also captures hospitalization details (admission/discharge dates and length of hospital stay), in-hospital mortality, medications, and interventional or surgical procedures classified under Japan’s original procedural codes. Diagnoses, comorbidities, and complications are coded using the International Classification of Diseases, Tenth Revision (ICD-10), along with additional text data in Japanese. However, laboratory data are not included in the database. Validation studies have demonstrated high sensitivity and specificity for the recorded diagnoses and procedures, confirming the reliability of the database for research purposes [14–16].

### Patient selection

We identified hospitalizations of patients aged ≥ 18 years with one of the following primary cancer diagnoses between July 1, 2010, and March 31, 2023: breast cancer (ICD-10 code, C50), colorectal cancer (C18, C19, C20), gastric cancer (C16), lung cancer (C34), and prostate cancer (C61) because they were the most prevalent cancers globally and in Japan; they accounted for 64% of all cancer cases in Japan [1, 17].

Hospitalizations with an unknown Tumor Node Metastasis (TNM) classification were excluded. The following baseline characteristics were included: age, sex, cancer stage**,** metastasis site, cause of hospitalization, disease status (initial diagnosis and recurrence), comorbidities (atrial fibrillation, chronic lung disease, chronic kidney diseases, chronic heart failure, diabetes, dyslipidemia, hypertension, ischemic heart diseases, liver disease, and stroke), anticancer agents, analgesics (non-steroidal anti-inflammatory drugs and opioids), year of admission, in-hospital mortality, and length of hospital stay. Supplementary Table 1 shows the ICD-10 codes for the cancer and comorbidity diagnoses.

Kampo medicine use was determined from in-hospital prescription data.

Kampo use was defined as any prescription of the 149 Kampo medicines available in Japan during the study period. Patients who received at least one Kampo medicine were identified, and all Kampo medicines were ranked by in-hospital use frequency. The top five Kampo medicines were identified for the overall population and for subgroups based on primary cancer diagnosis and cause of hospitalization (i.e., chemotherapy, surgery, radiation therapy, and others). We identified the Top 5 Kampo medicines co-prescribed with major anticancer agents (taxane, oxaliplatin and other platinum-based agents, irinotecan) used across different cancer types and analyzed trends in their co-prescription patterns.

### Statistical analyses

Categorical variables are reported as numbers and percentages. Continuous variables are summarized as medians with interquartile ranges (IQRs). Trend analysis for all Kampo prescription patterns was performed using the Cochran–Armitage test. All analyses were conducted using Stata/SE, version 18.0(StataCorp, College Station, TX, USA).

## Results

### Study population characteristics

A total of 9,838,370 hospitalizations with cancer as the primary diagnosis were registered from 1,798 hospitals in the DPC database between 2010 and 2023. A total of 2,975,686 hospitalizations of patients with an unknown TNM classification were excluded. Among the 6,862,684 eligible hospitalizations, 30% were for lung cancer, 29% for colorectal cancer, 20% for gastric cancer, 14% for breast cancer, and 7.4% for prostate cancer. The median age was 70 years (IQR: 62–76), and 59% were male. Kampo medicines were administered to 13. 6% of hospitalized patients, particularly among older adults (≥ 75 years) and those with colorectal cancer. Among patients receiving irinotecan, 25.1% were administered Kampo medicines. The corresponding proportions were 19.4% for intravenous molecularly targeted agents and 18.9% for antimetabolites. In contrast, Kampo medicine use among patients receiving other anticancer drugs was approximately 10% (Table 1). Lung cancer accounted for the highest proportion of stage IV cases (52%), followed by colorectal (44%) and prostate cancer (33%), whereas breast cancer had the lowest (17%). When admissions categorized as “others” (44%) were excluded, chemotherapy was the most frequent cause of hospitalization (36%), and the in-hospital mortality rate was 6.0% (Table 2).

**Table 1:** Inpatient characteristics and proportion of Kampo medicine users

	Overall		Kampo medicines users		Proportion of Kampo medicine users (%)
	*n* = 6,862,684		*n* = 920,774		13.6
Age category, *n* (%)					
18–49	491,420	(7.2)	47,983	(5.2)	9.9
50–64	1,639,823	(24)	201,727	(22)	12.5
65–74	2,560,163	(37)	338,870	(37)	13.4
≥75	2,171,278	(32)	332,194	(36)	15.6
Male, *n* (%)	4,057,190	(59)	569,058	(62)	14.2
Primary cancer diagnosis, *n* (%)					
Breast cancer	963,049	(14)	58,287	(6.3)	6.1
Colorectal cancer	1,994,544	(29)	478,845	(52)	24.0
Gastric cancer	1,369,300	(20)	164,001	(18)	12.0
Lung cancer	2,024,767	(30)	175,769	(19)	8.7
Prostate cancer	511,024	(7.4)	43,872	(4.8)	8.6
Cancer stage, *n* (%)					
I-II	2,904,372	(42)	344,438	(22)	12.1
III	1,193,930	(17)	205,082	(40)	17.4
IV	2,728,382	(40)	371,254	(37)	13.8
Site of metastasis, *n* (%)					
Bone	66,311	(7.2)	541,043	(9.3)	10.3
Brain	35,897	(3.9)	311,194	(5.3)	10.9
Liver	134,901	(15)	631,828	(11)	17.6
Lung	63,357	(6.9)	310,308	(5.3)	16.9
Lymph node	43,014	(4.7)	296,096	(5.1)	12.7
Cause of hospitalization, *n* (%)					
Chemotherapy	2,455,619	(36)	346,277	(38)	14.3
Surgery	1,108,118	(16)	153,491	(17)	14.0
Radiation therapy	285,022	(4.2)	33,338	(3.6)	11.8
Others	3,013,925	(44)	387,668	(42)	13.1
Disease status, *n* (%)					
Initial diagnosis, *n* (%)	5,958,691	(87)	794,050	(86)	13.5
Recurrence	867,065	(13)	122,629	(13)	14.3
Missing	36,928	(0.5)	4,095	(0.4)	11.4
Comorbidities, *n* (%)					
Atrial fibrillation	113,417	(1.7)	16,877	(1.8)	14.9
Chronic heart failure	536,316	(7.8)	36,049	(3.9)	6.8
Chronic kidney diseases	101,074	(1.5)	16,215	(1.8)	16.3
Chronic lung diseases	228,834	(3.3)	68,646	(7.5)	30.4
Diabetes	1,074,425	(16)	164,266	(18)	15.5
Dyslipidemia	621,246	(9.1)	95,881	(10)	15.8
Hypertension	1,550,919	(23)	248,818	(27)	16.3
Ischemic heart diseases	363,971	(5.3)	57,878	(6.3)	16.1
Liver diseases	255,761	(3.7)	40,370	(4.4)	16.0
Stroke	89,108	(1.3)	14,355	(1.6)	16.3
Anticancer agents, *n* (%)					
Antimetabolites	835,907	(12.2)	156,044	(17)	18.9
Endocrine therapy	190,541	(2.8)	22,178	(2.4)	11.9
Immune checkpoint inhibitors	140,947	(2.1)	13,565	(1.5)	10.0
Intravenous molecularly targeted agents	705,956	(10)	136,831	(15)	19.4
Irinotecan	318,978	(4.6)	79,318	(8.6)	25.1
Oral molecularly targeted agents	122,322	(1.8)	12,478	(1.4)	10.4
Platinum-based agents	845,343	(12)	93,192	(10)	11.1
Taxane	499,852	(7.3)	61,264	(6.7)	12.4
Analgesics, *n* (%)					
Non-steroidal anti-inflammatory drugs	3,188,970	(46)	2,638,019	(45)	16.2
Opioids	2,999,114	(44)	2,447,785	(42)	17.1

**Table 2:** Inpatient characteristics and in-hospital outcomes, stratified by primary cancer diagnosis

	Breast cancer		Colorectal cancer		Gastric cancer		Lung cancer		Prostate cancer	
	*n* = 963,049		*n* = 1,994,544		*n* = 1,369,300		*n* = 2,024,767		*n* = 511,024	
Age, years (IQR)	60 (49–70)		70 (62–77)		72 (65–78)		71 (64–76)		71 (66–76)	
Age category, *n* (%)										
18–49	243,065	(25)	117,565	(5.9)	57,414	(4.2)	71,340	(3.5)	2,036	(0.4)
50–64	340,637	(35)	497,253	(25)	270,010	(20)	443,791	(22)	88,132	(17)
65–74	233,180	(24)	719,158	(36)	508,235	(37)	849,666	(42)	249,924	(49)
≥ 75	146,167	(15)	660,568	(33)	533,641	(39)	659,970	(33)	170,932	(33)
Male, *n* (%)	4,963	(0.5)	1,162,815	(58)	969,843	(71)	1,408,545	(70)	511,024	(100)
Cancer stage, *n* (%)										
I-II	695,792	(72)	649,587	(33)	733,145	(54)	568,113	(28)	293,825	(57)
III	104,233	(11)	477,175	(24)	167,842	(12)	395,389	(20)	49,291	(9.6)
IV	163,114	(17)	867,782	(44)	468,313	(34)	1,061,265	(52)	167,908	(33)
Site of metastasis, *n* (%)										
Bone	96,996	(10)	54,894	(2.8)	27,132	(2.0)	317,676	(16)	118,652	(23)
Brain	23,147	(2.4)	14,608	(0.7)	5,761	(0.4)	305,999	(15)	1,493	(0.3)
Liver	46,351	(4.8)	491,198	(25)	139,476	(10)	90,013	(4.4)	8,189	(1.6)
Lung	47,211	(4.9)	246,927	(12)	32,488	(2.4)	39,368	(1.9)	12,005	(2.3)
Lymph node	110,206	(11)	80,186	(4.0)	47,324	(3.5)	96,146	(4.7)	10,369	(2.0)
Cause of hospitalization, *n* (%)										
Chemotherapy	285,500	(30)	716,600	(36)	351,127	(26)	989,589	(49)	112,803	(22)
Surgery	508,428	(53)	292,384	(15)	217,695	(16)	16,629	(0.8)	72,982	(14)
Radiation therapy	21,228	(2.2)	20,022	(1.0)	8,935	(0.7)	184,601	(9.1)	50,236	(9.8)
Others	147,893	(15)	965,538	(48)	791,543	(58)	833,948	(9.1)	275,003	(54)
Disease status, *n* (%)										
Initial diagnosis	859,565	(89)	1,663,461	(83)	1,278,313	(93)	1,716,725	(85)	440,627	(86)
Recurrence	100,040	(10)	321,333	(16)	86,282	(6.3)	292,968	(14)	66,442	(13)
Missing	3,444	(0.4)	9,750	(0.5)	4,705	(0.3)	15,074	(0.7)	3,955	(0.8)
Comorbidities, *n* (%)										
Atrial fibrillation	4,441	(0.5)	34,242	(1.7)	27,350	(2.0)	41,463	(2.0)	5,921	(1.2)
Chronic lung disease	20,440	(2.1)	73,013	(3.7)	52,991	(3.9)	378,426	(19)	11,446	(2.2)
Chronic kidney diseases	5,693	(0.6)	30,823	(1.5)	24,569	(1.8)	32,175	(1.6)	7,814	(1.5)
Chronic heart failure	15,570	(1.6)	65,509	(3.3)	47,519	(3.5)	87,353	(4.3)	12,883	(2.5)
Diabetes	82,368	(8.6)	341,754	(17)	217,370	(16)	362,274	(18)	70,659	(14)
Dyslipidemia	61,939	(6.4)	182,765	(9.2)	120,225	(8.8)	220,063	(11)	36,254	(7.1)
Hypertension	116,077	(12)	517,621	(26)	321,101	(23)	508,390	(25)	87,730	(17)
Ischemic heart disease	17,359	(1.8)	107,706	(5.4)	89,527	(6.5)	124,446	(6.1)	24,933	(4.9)
Liver disease	26,359	(2.7)	82,963	(4.2)	57,574	(4.2)	76,107	(3.8)	12,758	(2.5)
Stroke	4,342	(0.5)	25,516	(1.3)	21,846	(1.6)	31,880	(1.6)	5,434	(1.1)
Anticancer treatments, *n* (%)										
Antimetabolites	44,327	(4.6)	549,187	(28)	14,425	(1.1)	227,537	(11)	431	(0.1)
Endocrine therapy	72,074	(7.5)	12,272	(0.6)	8,910	(0.7)	14,059	(0.7)	83,226	(16)
Immune checkpoint inhibitors	912	(0.1)	718	(0.04)	12,271	(0.9)	126,977	(6.3)	49	(0.01)
Intravenous molecularly targeted agents	90,587	(9.4)	446,377	(22)	64,144	(4.7)	104,845	(5.2)	3	(0.001)
Irinotecan	647	(0.1)	245,141	(12)	20,783	(1.5)	51,565	(2.5)	842	(0.2)
Oral molecularly targeted agents	2,301	(0.2)	5,119	(0.3)	413	(0.03)	114,363	(5.6)	126	(0.0)
Platinum-based agents	3,484	(0.4)	3,577	(0.2)	178,536	(13)	653,712	(32)	6,034	(1.2)
Taxane	122,635	(13)	330	(0.02)	74,207	(5.4)	249,049	(12)	53,631	(10)
Analgesics, *n* (%)										
Non-steroidal anti-inflammatory drugs	626,457	(65)	924,525	(46)	490,306	(36)	904,446	(45)	243,236	(48)
Opioids	560,999	(58)	971,176	(49)	522,946	(38)	708,867	(35)	235,126	(46)
In hospital mortality, *n* (%)	28,095	(2.9)	94,767	(4.8)	88,102	(6.4)	178,244	(8.8)	21,283.0	(4.2)
Length of hospital stay, days (IQR)	7.0 (3.0–10.0)	(2.9)	10.0 (3.0–19.0)	(4.8)	10.0 (6.0–18.0)	(6.4)	11.0 (5.0–19.0)	(8.8)	9.0 (2.0–14.0)	(4.2)

*IQR* interquartile range

### Trends in Kampo medicine prescriptions

**Fig. 1:**
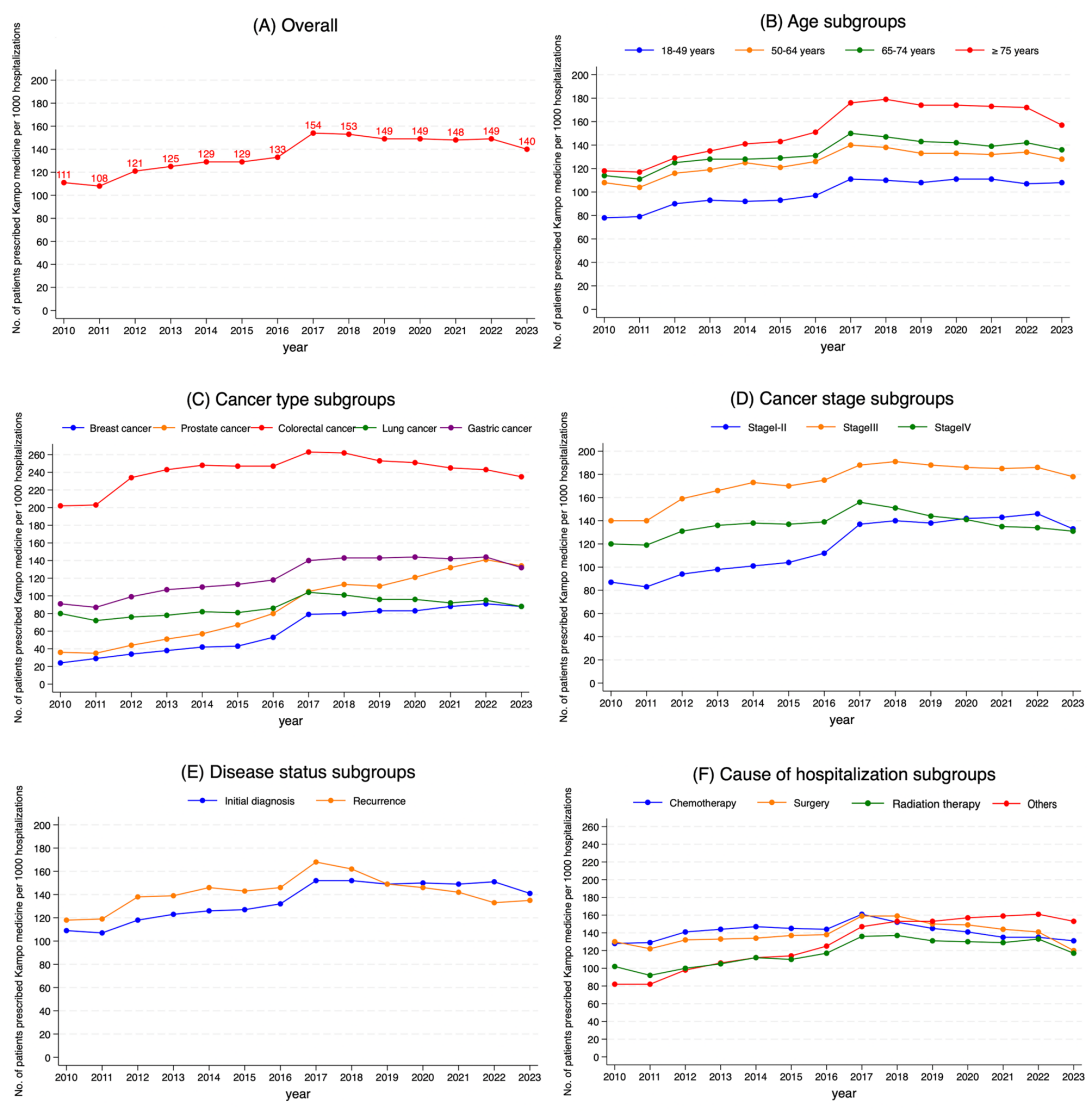
Fourteen-year trends in use in the overall population and clinically relevant subgroups. **A** Overall, **B** age, **C** cancer type, **D** cancer stage, **E** disease status, and **F** cause of hospitalization subgroups

Kampo medicine prescriptions increased from 111 per 1,000 hospitalizations in 2010 to a peak of 154 per 1,000 in 2017, followed by a slight decline to 140 per 1,000 in 2023 (Cochran–Armitage test, *P* < 0.001) (Fig. 1A). When stratified by age group, Kampo prescriptions were consistently more frequent among older adults, particularly those aged ≥ 75 years. The prescription proportion for breast cancer increased from 24 per 1,000 hospitalizations in 2010 to 88 per 1,000 in 2023. For prostate cancer, the proportion increased from 36 per 1,000 in 2010 to 134 per 1,000 in 2023. This represents a 3.7-fold increase for both cancers. The prescription proportion for colorectal cancer was the highest among all cancer types, increasing from approximately 200 per 1,000 hospitalizations in 2010 to a peak of approximately 260 per 1,000 in 2017, then gradually declining to 230 per 1,000 in 2023 (Fig. 1C). When categorized by reason for hospitalization, Kampo prescriptions peaked in 2017 for both chemotherapy and surgery admissions, followed by a decline. In contrast, prescriptions for “other” hospitalizations increased by approximately 1.8-fold from 2010 to 2023 (Fig. 1F).

### Top five Kampo medicines

**Fig. 2:**
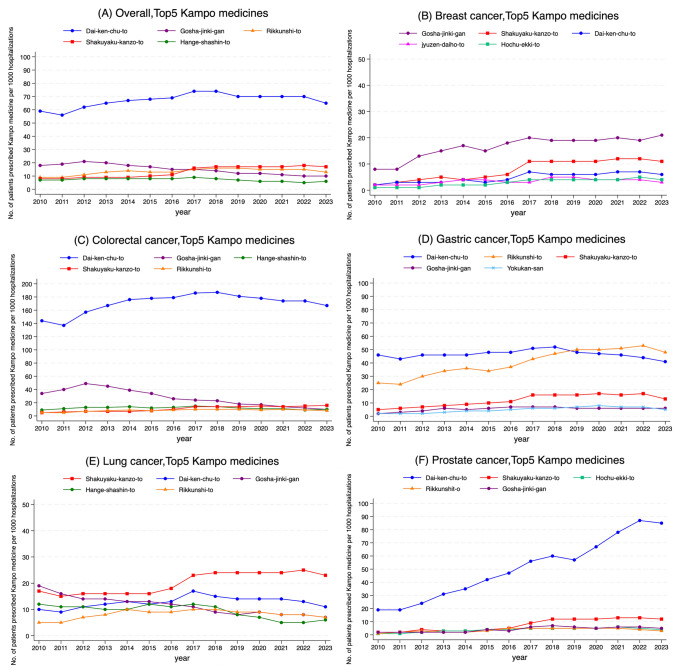
Fourteen-year trends for the top five Kampo medicines in the overall study population and cancer type subgroups. **A** Overall, **B** breast, **C** colorectal, **D** gastric, **E** lung, and **F** prostate cancers

**Fig. 3:**
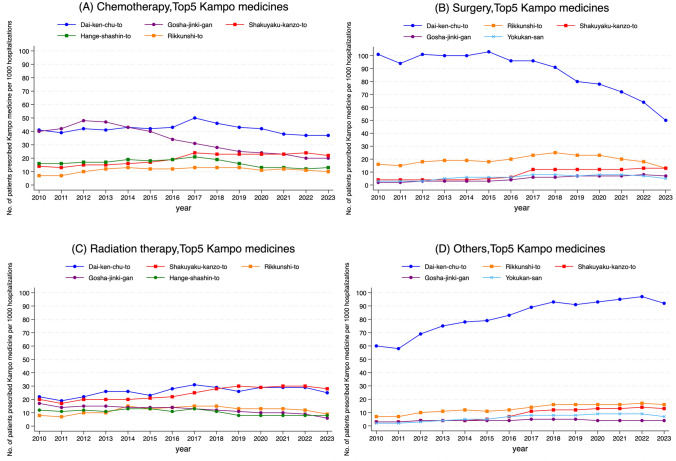
Fourteen-year trends for the top five Kampo medicines in the cause of hospitalization subgroups. **A** Chemotherapy, **B** surgery, **C** radiation therapy, and **D** others

The five most frequently prescribed Kampo medicines were Dai-ken-chu-to, Gosha-jinki-gan, Rikkunshi-to, Shakuyaku-kanzo-to, and Hange-shashin-to (Fig. 2A). Dai-ken-chu-to was the most prescribed and remained stable after peaking in 2017. In surgical hospitalizations, its prescription proportion declined after 2015, reaching approximately half of the 2015 level by 2023 (Fig. 3B). In contrast, its use in other hospitalizations increased approximately 1.5 times in 2023 compared with 2010. In prostate cancer, it increased approximately 4 times over the same period (Fig. 2F and 3D). The use of Gosha-jinki-gan peaked in 2012 and declined thereafter. A comparable trend was observed in patients with colorectal cancer (Fig. 2C) and in chemotherapy-related hospitalizations (Fig. 3A). In contrast, the use of Gosha-jinki-gan continued to increase gradually after 2012 among patients with breast cancer (Fig. 2B).

### Top 5 Kampo medicines and prescription patterns with major anticancer agents

**Fig. 4:**
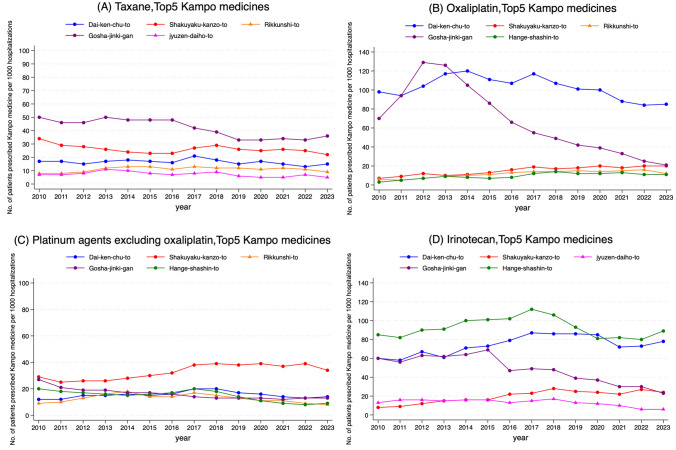
Fourteen-year trends for the top five Kampo medicines in specific chemotherapy subgroups. **A** Taxane, **B** oxaliplatin, **C** platinum agents excluding oxaliplatin, and **D** irinotecan

For patients treated with taxane, Gosha-jinki-gan was the most frequently prescribed Kampo medicine among the top five Kampo medicines, with its prescription rate gradually decreasing after 2016 (Fig. 4A). For patients treated with oxaliplatin, the proportion of Gosha-jinki-gan prescriptions peaked in 2012 and then sharply declined, reaching approximately one-sixth of the peak level by 2023. (Fig. 4B).

## Discussion

This nationwide study analyzed data from 6,862,684 hospitalizations of patients with cancer in Japan between 2010 and 2023 to examine trends in Kampo medicine use in real-world clinical practice. Kampo medicines were prescribed in 13.6% of hospitalizations and were more frequently used among older adults and patients with colorectal cancer. The overall prescription proportion increased until 2017 and then gradually declined thereafter. In breast and prostate cancers, the proportion had increased approximately fourfold by 2023. The five most frequently prescribed Kampo medicines were Dai-ken-chu-to, Gosha-jinki-gan, Rikkunshi-to, Shakuyaku-kanzo-to, and Hange-shashin-to. Dai-ken-chu-to was the most commonly prescribed throughout the study period.

Kampo prescriptions were more prevalent in patients with Stage III–IV than in the overall patients (77% vs. 57%). These results suggest that Kampo may play a crucial role for inpatients who are severely ill.

The changes in Kampo prescription patterns may be attributed to recent shifts in the treatment environment, such as the spread of evidence-based clinical guidelines [18] and the wider use of new supportive care drugs [19–21]. Kampo medicines were more frequently prescribed to older patients. This likely reflects their role in relieving symptoms related to aging and cancer treatment. Common symptoms, such as appetite loss, constipation, and fatigue, are frequently observed in older patients with cancer. Standard treatments do not always effectively manage these symptoms [22]. Prescription patterns also varied according to the reason for hospitalization. Prescriptions for chemotherapy or surgery peaked in 2017 and then decreased. In contrast, prescriptions for other types of hospitalizations continued to increase. These trends suggest that Kampo medicines are now used not only for acute symptoms but also as supportive care for managing chronic symptoms and improving general health [7, 9].

Dai-ken-chu-to, Gosha-jinki-gan, Rikkunshi-to, Shakuyaku-kanzo-to, and Hange-shashin-to are commonly used as supportive care for managing a variety of symptoms associated with cancer treatment. Dai-ken-chu-to is used for symptoms such as reduced intestinal motility, postoperative inflammation following gastrectomy colorectal surgery, and radiation-induced enteritis [8]. Gosha-jinki-gan is used for chemotherapy-induced peripheral neuropathy [23]. Rikkunshi-to is used for appetite loss, Shakuyaku-kanzo-to for muscle cramps, and Hange-shashin-to for oral mucositis and diarrhea [7, 24]. These five Kampo medicines were also ranked among the top five in a previous study that analyzed their use during chemotherapy across various cancer types [25].

Randomized controlled trials have demonstrated that Dai-ken-chu-to reduces the risk of postoperative bowel obstruction after abdominal surgery [26, 27]. In the present study, Dai-ken-chu-to was the most frequently prescribed Kampo medicine; however, its use varied depending on cancer type and reason for hospitalization. Prescriptions for surgical admissions peaked in 2015 and then declined. In contrast, prescriptions for “other” admissions continued to increase. This trend suggests a shift in the use of Dai-ken-chu-to from acute postoperative management to supportive care in chronic settings, such as improving digestive symptoms and general health. Advances in medical techniques may explain the decrease in postoperative use. In particular, widely adopted minimally invasive surgeries, such as thoracoscopic and robot-assisted procedures, have been reported to reduce the risk of postoperative bowel dysfunction and obstruction [28]. This change may have contributed to the reduced postoperative use of Dai-ken-chu-to. On the other hand, Dai-ken-chu-to has been reported to be effective for constipation, gastrointestinal symptoms related to constipation, and fecal incontinence in older patients [20, 29]. Its use may have expanded for symptom relief. Prostate cancer is common in older adults, and treatment-related symptoms, such as constipation and abdominal discomfort, are also frequent [30]. Moreover, since 2000, the number of patients with prostate cancer aged ≥ 70 years has increased markedly, reflecting the aging of this patient population [31]. These factors may help explain the increase in Dai-ken-chu-to prescriptions among patients with prostate cancer.

Gosha-jinki-gan has been used for the prevention and relief of chemotherapy-induced peripheral neuropathy (CIPN) in Japan [7]. However, in the present study, its prescriptions peaked in 2012 and subsequently declined, particularly among patients with colorectal cancer and those hospitalized for chemotherapy. The decline was evident in patients receiving oxaliplatin. This finding may suggest a strong association with specific chemotherapy protocols. In colorectal cancer, the FOLFOX regimen, which includes oxaliplatin, is widely used [32]. Randomized controlled trials have not demonstrated the preventive efficacy of Gosha-jinki-gan for oxaliplatin-induced CIPN [33–35]. The American Society of Clinical Oncology published its first guideline on CIPN in 2014 [36], In the 2020 update [18]. duloxetine remained the only recommended agent, while other treatments were not supported by sufficient evidence. Similarly, the Japanese guideline on CIPN does not recommend the use of Gosha-jinki-gan for the prevention of oxaliplatin-induced CIPN [37]. These accumulated negative findings and changes in clinical guidelines may have contributed to the decreased use of Gosha-jinki-gan among patients with colorectal cancer and those receiving inpatient chemotherapy. In contrast, despite insufficient evidence supporting the efficacy, Gosha-jinki-gan was administered to inpatients treated with taxane presumably for CIPN management. Notably, prescriptions of Gosha-jinki-gan increased after 2012 in patients with breast cancer, who are commonly treated with taxane-based regimens. Previous studies have reported that younger breast cancer survivors often experience long-term impairment in health-related quality of life even years after treatment [38]. Therefore, the increasing prescriptions may reflect clinical efforts to manage chronic CIPN and to improve their quality of life. For taxane-induced CIPN in breast cancer, a small randomized controlled trial has suggested the potential efficacy of Gosha-jinki-gan [39]. and its use may have continued or increased. Nevertheless, two meta-analyses targeting CIPN associated with various anticancer agents did not consistently confirm its efficacy [23, 40]. and the overall evidence remains limited. More recently, a randomized controlled trial reported a preventive effect of Gosha-jinki-gan in a paclitaxel-based regimen for ovarian cancer [41]. The findings suggest its potential efficacy against taxane-induced neuropathy. Nonetheless, careful interpretation is warranted given differences in cancer types and treatment settings. Further evidence is needed to evaluate the efficacy of Gosha-jinki-gan, specifically in patients receiving paclitaxel.

These findings also highlight the clinical and epidemiological value of examining long-term trends in Kampo medicine use among acutely hospitalized patients with cancer. Clinically, to clarify the needs and challenges related to symptom management in acute cancer care, it is important to understand the pattern of Kampo prescription as supportive therapies in inpatients requiring acute care. Epidemiologically, researchers can evaluate changes in medical practice resulting from shifts in healthcare systems, treatment guidelines, and therapeutic strategies, analyzing long-term usage trends.

This study has several limitations. First, the DPC database includes only inpatient information. Therefore, Kampo use in outpatient settings and over the long term could not be assessed. Second, the database lacks subjective information, such as the reasons for prescriptions and patient symptoms, which limits the interpretation of the results.

This nationwide cohort study showed that the 14-year prescription patterns of Kampo medicines in patients with cancer in Japan changed over time and varied depending on clinical settings. The overall prescription rate increased from 2010 to 2017 before plateauing. However, the trends varied across age group, cancer type, cancer stage, disease status, and cause of hospitalization. These findings suggest that Kampo medicines have been used selectively as supportive care based on the clinical situation of each patient.

## Data Availability

The data analyzed during the current study are not publicly available due to contracts with the hospitals providing data to the database. Further inquiries on data can be directed to the corresponding author.
